# The Chemopreventive Properties and Therapeutic Modulation of Green Tea Polyphenols in Oral Squamous Cell Carcinoma

**DOI:** 10.5402/2011/403707

**Published:** 2011-05-16

**Authors:** Ui-Lyong Lee, Sung-Weon Choi

**Affiliations:** ^1^Tooth Bioengineering National Research Lab, BK21, and Dental Research Institute, School of Dentistry, Seoul National University, Seoul 110-749, Republic of Korea; ^2^Department of Oral & Maxillofacial Surgery, Hangang Sacred Heart Hospital, Hallym University, Seoul 140-719, Republic of Korea; ^3^Oral Oncology Clinic, Research Institute and Hospital, National Cancer Center, 323 Ilsan-ro, Ilsandong-gu, Goyang-si, Gyeonggi-do 410-769, Republic of Korea

## Abstract

Chemoprevention is a relatively novel and promising approach for controlling cancer that uses specific natural products or synthetic agents to suppress, reverse, or prevent premalignancy before transformation into invasive cancer. Oral cavity squamous cell carcinoma (OCSCC) represents a large, worldwide health burden with approximately 274,000 cases diagnosed annually worldwide. Smoking and alcohol consumption are major inducers of OCSCC. Recently, the human papilloma virus was also shown to potentially be an etiologic factor. Due to its easily identifiable risk factors and the presence of premalignant regions, oral cancer makes a good candidate for chemoprevention. Green tea is the most widely consumed beverage in the world, and it has received considerable attention because of its abundant, scientifically proven, beneficial effects on human health. In this review, we discuss the role of green tea in oral cancer chemoprevention with regard to the multiple molecular mechanisms proposed in various in vitro, in vivo, and clinical trials.

## 1. Introduction

Oral squamous cell carcinoma represents a large, worldwide health burden as the eleventh most common cancer type with approximately 274,000 cases diagnosed annually worldwide [1, 2]. Carcinogenesis is a multistage process consisting of initiation, promotion, and progression phases [3]. Thus, the multistage sequence of events has many phases for prevention and intervention. Despite therapeutic advances in this disease, OCSCC is associated with severe morbidity with a 5-year overall survival rate of less than 50% [4] and a high occurrence of second primary tumors (20% to 30%) [5–7]. The overall 5-year survival rate of OCSCC has not significantly improved in the past 30 years [2]. Smoking and alcohol consumption are major inducers of OCSCC. The prevalence of OCSCC in smokers is 4–7 times higher than in nonsmokers, and if alcohol or chewing tobacco is augmented to the cigarette usage, the incidence of OCSCC is increased by 19- and 123-fold, respectively [8]. In recent years, the human papilloma virus (HPV), particularly HPV type 16, has also been suggested as an etiologic factor for OCSCC, especially among younger patients who are nonsmokers with no or minimal alcohol consumption [9, 10]. Patients diagnosed with OCSCC who have been continually exposed to smoking and alcohol are at a high risk for recurrences and second primary tumors (SPTs) [2]. 

Chemoprevention, a notion introduced by Sporn et al. [11], is the use of natural products or synthetic chemicals for the reversal, suppression, or prevention of the premalignant transformation of cells to a malignant form. In this respect, 13-cis- retinoic acid has been reported to be effective in the prevention of transformation of oral leukoplakia and dysplasia [12] and to stop the development of second primary tumors in patients with head and neck squamous cell carcinoma [5, 6]. However, critical problems with retinoids include toxicity and the recurrence of the leukoplakia after the cessation of treatment [12]. Thus, the investigation for a better chemopreventive agent is warranted. 

An ideal chemopreventive agent must have (i) little or no toxicity, (ii) a high efficacy in multiple sites, (iii) the capability for oral consumption, (iv) a known mechanism of action, (v) low cost, and (vi) human acceptance [13]. Currently, natural products, especially the antioxidants present in common food and beverages, have obtained great attention for cancer prevention owing to their various health benefits, noticeable lack of toxicity/side effects, and the limitations of other chemotherapeutic agents [14]. Several studies have shown that natural products composed of a wide spectrum of biologically active phytochemicals, including phenolics, flavonoids, carotenoids, alkaloids, and nitrogen, suppress the early and late stages of carcinogenesis [15]. 

Green tea is the most widely consumed beverage in the world and is especially popular in the Far East. Because of its abundant, scientifically proven, beneficial effects on human health, green tea has received considerable attention [16]. The polyphenols found in green tea have been documented to inhibit a variety of processes associated with cancer cell growth, survival, and metastasis [17]. 

Here, we highlight the evidence of green tea's role in oral cancer chemoprevention with regard to multiple molecular mechanisms proposed in various in vitro, in vivo, and clinical trials.

## 2. Green Tea and Polyphenols

Green teas are derived from *Camellia sinensis* and represent the most consumed beverages in the world, next to water [18]. Green tea contains polyphenols, glycosides, leucoanthocyanins, and phenol acid [19], with the polyphenols constituting 36% of the fresh green tea leaf dry weight [20]. 

In a randomized controlled trial (RCT), green tea was documented to significantly reduce the level of total serum cholesterol and low density lipoprotein (LDL) cholesterol in the green tea group, which was treated with 150 mg green tea catechins and 150 mg of other tea polyphenols a day for 3 months [21]. A meta-analysis showed that an increase in tea consumption of three cups (711 mL/day) decreased the risk of a myocardial infarction by 11% [22]. Epidemiological studies have also shown that the intake of green tea may reduce the risk of malignancy [23, 24]. In 1997, Imai and colleagues published a prospective cohort survey of more than 8,552 Japanese individuals that demonstrated the ingestion of more than 10 daily cups (120 mL each) of green tea decreased the development of cancer [25]. Additionally, in 2009, Myung and colleagues performed a meta-analysis of epidemiologic studies that found green tea consumption to have a preventative effect on stomach cancer [26]. Seely et al. also reported that green tea consumption may possibly help prevent breast cancer recurrence in early-stage (I and II) cancers [27]. In many mouse skin tumor models, protection against UVB-induced skin carcinogenesis and the inflammatory response was obtained with topical application or oral consumption of green tea [28–31]. 

Green tea contains four major polyphenols: epicatechin (EC), epigallocatechin (EGC), epicatechin-3-gallate (ECG), and epigallocatechin-3-gallate (EGCG), composing 1–3%, 3–6%, 3–6%, and 7–13%, respectively, of the fresh green tea leaf dry weight [20, 32]. Numerous studies have reported that green tea polyphenols have plentiful beneficial effects on human health, including not only having antiviral, anti-inflammatory, and antiallergic effects, but also aiding in weight control [33, 34]. It has also been documented that polyphenols inhibit tumorigenesis in a variety of organs, including skin, lung, oral cavity, esophagus, stomach, small intestine, colon, liver, pancreas, ovary, and mammary gland [35–39]. 

The polyphenol EGCG has specifically been documented to have chemopreventive effects against various cancers [40–45], and one cup of green tea holds 200 mg of EGCG [46]. EGCG has been reported to have multiple biologic functions, including preventing the formation of blood vessels (angiogenesis) and organizing blood vessel permeability to hinder the blood supply to cancerous cells [47, 48]. Adding EGCG to cells leads to decreased phosphorylation of the epidermal growth factor receptor (EGFR), Akt, and Stat3, without regulating protein levels [44, 49]. EGCG has also been shown to inhibit telomerase activity and lead to telomerase fragmentation [50]. Green tea polyphenols regulate multiple points controlling cancer cell growth, survival, and metastasis, with effects at the DNA, RNA, and protein levels [18]. 

Desirable green tea ingestion is known to be 3 to 5 cups per day (up to 1200 mL/day), providing a minimum of 250 mg/day catechins [51].

## 3. The Chemopreventive Effect of Green Tea Polyphenols on Oral Cancer: In Vitro Studies

Numerous in vitro studies have shown the antiproliferative effect and apoptosis inducing capability of green tea polyphenols in oral cancer cell lines. Jeffrey et al. have reported that the antiproliferative effects of green tea polyphenols and EGCG were more noticeable towards oral cancer cell lines (CAL27, HSC-2, and HSG1) than normal fibroblasts (GN56 and HGF-1) [52]. The application of EGCG to cell culture medium resulted in the formation of hydrogen peroxide. This result was consistent with EGCG performing as a pro-oxidant because tumor cells are known to be more vulnerable to oxidative forces than normal cells [52, 53].An in vitro, multistage tumorigenesis model for oral cancer showed that cell proliferation was arrested at all stages of carcinogenesis by EGCG with the best efficacy demonstrated in dysplastic cells [54]. A study by Masuda and coworkers [49] demonstrated that treatment with EGCG increased the proportion of cells in the G_1_ phase of the cell cycle and induced apoptosis. Moreover, in cells treated with EGCG, a reduction in the cyclin D1 protein and in the hyperphosphorylated form of pRB was observed [49]. Liu et al. showed that green tea extract and epigallocatechin-3 gallate inhibited the growth of 3 squamous cell lines (CAL-27, SCC-25, and KB) via S and G_2_/M phase arrest. The major signaling cascades influenced by green tea extract and EGCG were shown to be the EGFR and Notch pathways, which in turn, affected cell-cycle-related networks [55]. 

Hsu et al. reported that treatment of caspase 3 wild-type oral carcinoma cell lines with EGCG resulted in a gradual decrease of mitochondrial function down to an insignificant level, but caspase 3 null cells did not undergo apoptosis. This result demonstrates that green tea polyphenol-associated apoptosis is mitochondria targeted and caspase 3 dependent [56]. EGCG was also found by cDNA microarray to upregulate p21WAF1 in the OSC2 oral cancer cell line, which may facilitate caspase 3-mediated apoptosis [57]. 

The overexpression of HER-2 (neu/erbB2) is correlated with a poor prognosis in patients with breast cancer or oral squamous cell carcinoma, presumably due to an increased metastatic ability and resistance to various cancer chemotherapies [58, 59]. Masuda et al. [45] reported that treatment of head and neck squamous cell carcinoma cell lines (HNSCC) (YCU-H891, YCU-N861) with EGCG resulted in a 50% inhibition of growth and a marked inhibition of HER-2 phosphorylation. This outcome was connected with the prevention of Stat3 activation as well as the inhibition of c-fos and cyclin D1 promoter activity. Recent studies have also demonstrated that RECK methylation is associated with enhancing metastasis and invasion in human cancers [60–62]. 

Chen et al. illustrated that the invasion, motility, migration, and secretion of MMP-2 and u-PA in SCC-9 oral cancer cells, through attenuation of p-FAK and p-Src, could be significantly inhibited by epigallocatechin-3 gallate [63]. Long et al. reported that hypermethylation of the RECK gene is associated with a poor prognosis in oral squamous cell carcinoma [64]. A study by Kato et al. showed the treatment of oral cancer cells with EGCG partially reversed the hypermethylation status of the RECK gene and significantly increased the expression level of RECK mRNA [65]. EGCG was also found to reduce the expression of MMP-2 and MMP-9 [16, 65]. Taken together, these outcomes demonstrate that green tea polyphenols may reduce the invasion and migration of human oral cancer cells. 

The concept of combination chemoprevention pursues the ability to enhance the chemopreventive efficacy of both agents (synergism), while reducing side effects by dose reduction. Synergistic interactions were shown between beta-carotene and alpha-tocopherol in an in vivo hamster cheek pouch tumorigenesis model [66] as well as between beta-carotene and anticancer alkylating agents in an in vitro study that utilized tongue squamous carcinoma cells [67]. Khafif et al. documented an interactive synergistic effect of EGCG and curcumin treatment. Specifically, EGCG arrested malignant human oral epithelial cells in G_1_, whereas curcumin blocked cells in S/G_2_M [68]. The potency of the cytotoxic and apoptotic effects on human tongue squamous carcinoma cells was greater with a combination of lacroferrin and tea polyphenols than with polyphenols alone [69]. A study by Amin et al. showed that EGCG and erlotinib (epidermal growth factor receptor tyrosine kinase inhibitor) had a synergistic growth-inhibitory effect in a nude mouse xenograft model of squamous cell carcinomas of the head and neck via inhibition of the nuclear factor-*κ*B signaling pathway [70]. EGCG was found to protect normal salivary gland cells from the effects of gamma irradiation and the chemotherapy drug cis-platinum(II)diammine dichloride (CDDP), suggesting that the combination of green tea consumption with chemotherapy or radiotherapy could be a promising avenue of treatment [71].

## 4. The Chemopreventive Effect of Green Tea Polyphenols on Oral Cancer: In Vivo Studies

Experimental carcinogenesis models are valuable tools to investigate the multistep characteristics of carcinogenesis and to study various modulations that intervene in the development of cancer. These models also have many advantages over simple in vivo studies. The hamster buccal pouch carcinogenesis model (HBP) has been extensively employed to investigate the chemopreventive effectiveness of medical plants and dietary agents [72]. Treatment with 7,12-dimethylbenz[a]anthracene (DMBA) induced HBP carcinoma and upregulated genotoxicity. Inhibition of DMBA-induced HBP carcinomas by green tea polyphenols was observed, and polyphenol treatment was associated with a significant reduction in phase I enzymes, regulation of lipid peroxidation, and increased antioxidant and phase II enzyme activities in this model [73]. DMBA-induced oral carcinogenesis was arrested by EGCG in hamsters showing a lower risk of dysplasia and oral carcinoma [74]. 4-nitroquinoline 1-oxide (4-NQO) is also known to induce multistep carcinogenesis [75]. Srinivasan et al. reported a significant reduction in the number of tumors, tumor volume, and oral squamous cell carcinomas in green tea polyphenol treated rats relative to 4-NQO induced animals. Additionally, application of green tea polyphenols in this study was shown to enhance the activity of phase II enzymes (glutathione-S-transferase and UDP-glucuronyl transferase) and reduce the activity phase I enzymes (cytochrome b5, cytochrome P450, cytochrome b5 reductase, cytochrome P450 reductase, aryl hydrocarbon hydroxylase, and DT-diaphorase) [76]. EGCG was also sufficient to stop phorbol-12-myristate-induced cell invasion and matrix metalloproteinase-9 expression, as demonstrated by the inhibition of tumor growth observed with EGCG treatment in SCC-9 cells in vivo via a cancer cell xenograft nude mouse model [63]. 

Recent studies have also reported that amyloid precursor protein (APP) is upregulated in pancreatic cancer cells and SW837 colon carcinoma cells both in vitro and in vivo [77, 78]. Ko et al. demonstrated that APP was significantly upregulated in MBN-induced HBP carcinomas but was significantly decreased by tea intake [79]. Moreover, APP expression and secretion from oral squamous cell carcinoma was arrested by the application of green tea polyphenols in a dose-dependent manner [79].

## 5. The Chemopreventive Effect of Green Tea Polyphenols on Oral Cancer: Clinical Trials and a Prospective Cohort Study

Schwartz et al. performed a pilot study in which green tea total extracts (2000–2500 mg/day) were administrated in drinking water to smokers for four weeks [80]. The study found that during the course of green tea administration, smoking-induced DNA damage was reduced, cell growth was inhibited, the percentage of cells in S phase was decreased, cells accumulated in G_1_ phase, DNA content became more diploid and less aneuploid, and markers of apoptosis were upregulated [80]. A double-blind intervention trial was performed in patients with oral mucosa leukoplakia using mixed tea extracts [81]. After 6 months, the oral leukoplakia was reduced in size in 40% of the patients who ingested tea extracts. In a phase II randomized, placebo-controlled clinical trial of green tea extract (500, 750, or 1,000 mg/m^2^) in high risk oral premalignant lesions (OPL), the OPL clinical response rate was higher in all green tea extract (GTE) arms versus placebo but did not reach statistical significance [82]. However, higher-dose GTE arms displayed higher responses and demonstrated a dose-dependent effect [82]. Moreover, stromal VEGF and cyclin D1 expressions were reduced in clinically responsive GTE patients and increased in nonresponsive patients at 12 weeks [82].

Ide and colleagues conducted a prospective nationwide, large-scale cohort study in Japan. A total of 20,550 men and 29,671 women aged 40–79 years without any history of oral cancer were included [83]. During a mean follow-up period of 10.3 years, 37 oral cancer cases were identified. For women, the hazard ratios (HRs) of oral cancer for a green tea consumption of 1-2, 3-4, or 5 or more cups per day, were 0.51, 0.60, and 0.31, respectively, when compared with individuals who drank less than one cup per day. However, for men, no such trends were identified [83]. 

## 6. Conclusion

This paper summarized the chemopreventive efficacy of green tea polyphenols in various in vitro and in vivo oral cancer models. All in vitro and in vivo studies demonstrated that green tea can regulate numerous molecular pathways involved in cancer promotion and progression. The epidemiology and multistep carcinogenesis of oral cancer suggests that it is a preventable cancer. Prevention is the most ideal strategy to save lives and reduce the morbidity of radical surgery. Although small trials have shown promising results, chemoprevention clinical trials with green tea performed in oral cancer are very limited. Substantial clinical trials are required to investigate the chemopreventive effectiveness of green tea polyphenols against oral cancer either alone or in combination with other chemopreventive agents. To seek a fundamental association between the consumption of green tea and a reduced rate of oral cancer development or mortality, more randomized clinical trials (RCTs) and cohort studies are needed. 

## References

[1] Parkin DM, Bray F, Ferlay J, Pisani P (2005). Global cancer statistics, 2002. *CA: Cancer Journal for Clinicians*.

[2] Kupferman ME, Myers JN (2006). Molecular biology of oral cavity squamous cell carcinoma. *Otolaryngologic Clinics of North America*.

[3] Vincent TL, Gatenby RA (2008). An evolutionary model for initiation, promotion and progression in carcinogenesis. *International Journal of Oncology*.

[4] Fuller CD, Wang SJ, Thomas CR (2007). Conditional survival in head and neck squamous cell carcinoma: results from the SEER dataset 1973–1998. *Cancer*.

[5] Benner SE, Pajak TF, Lippman SM (1994). Prevention of second primary tumors with isotretinoin in patients with squamous cell carcinoma of the head and neck: long-term follow-up. *Journal of the National Cancer Institute*.

[6] Hong WK, Lippman SM, Itri LM (1990). Prevention of second primary tumors with isotretinoin in squamous-cell carcinoma of the head and neck. *The New England Journal of Medicine*.

[7] McDonald S, Haie C, Rubin P (1989). Second malignant tumors in patients with laryngeal carcinoma: diagnosis, treatment, and prevention. *International Journal of Radiation Oncology Biology Physics*.

[8] Ko YC, Huang YL, Lee CH (1995). Betel quid chewing, cigarette smoking and alcohol consumption related to oral cancer in Taiwan. *Journal of Oral Pathology and Medicine*.

[9] Fakhry C, Gillison ML (2006). Clinical implications of human papillomavirus in head and neck cancers. *Journal of Clinical Oncology*.

[10] Tran N, Rose BR, O’Brien CJ (2007). Role of human papillomavirus in the etiology of head and neck cancer. *Head and Neck*.

[11] Sporn MB, Dunlop NM, Newton DL, Smith JM (1976). Prevention of chemical carcinogenesis by vitamin A and its synthetic analogs (retinoids). *Federation Proceedings*.

[12] Hong WK, Endicott J, Itri LM (1986). 13-cis-retinoic acid in the treatment of oral leukoplakia. *The New England Journal of Medicine*.

[13] Rajamanickam S, Agarwal R (2008). Natural products and colon cancer: current status and future prospects. *Drug Development Research*.

[14] Manson MM, Farmer PB, Gescher A, Steward WP (2005). Innovative agents in cancer prevention. *Recent Results in Cancer Research*.

[15] Nishino H, Satomi Y, Tokuda H, Masuda M (2007). Cancer control by phytochemicals. *Current Pharmaceutical Design*.

[16] Ho YC, Yang SF, Peng CY (2007). Epigallocatechin-3-gallate inhibits the invasion of human oral cancer cells and decreases the productions of matrix metalloproteinases and urokinase-plasminogen activator. *Journal of Oral Pathology and Medicine*.

[17] Gu Q, Hu C, Chen Q (2008). Prevention of chinese green tea on 3,4-benzopyrene-induced lung cancer and its mechanism in animal mode. *Zhongguo Fei Ai Za Zhi*.

[18] Beltz LA, Bayer DK, Moss AL, Simet IM (2006). Mechanisms of cancer prevention by green and black tea polyphenols. *Anti-Cancer Agents in Medicinal Chemistry*.

[19] Balentine DA, Wiseman SA, Bouwens LC (1997). The chemistry of tea flavonoids. *Critical Reviews in Food Science and Nutrition*.

[20] Graham HN (1992). Green tea composition, consumption, and polyphenol chemistry. *Preventive Medicine*.

[21] Maron DJ, Lu GP, Cai NS (2003). Cholesterol-lowering effect of a theaflavin-enriched green tea extract: a randomized controlled trial. *Archives of Internal Medicine*.

[22] Peters U, Poole C, Arab L (2001). Does tea affect cardiovascular disease? A meta-analysis. *American Journal of Epidemiology*.

[23] Ji BT, Chow WH, Hsing AW (1997). Green tea consumption and the risk of pancreatic and colorectal cancers. *International Journal of Cancer*.

[24] Yu GP, Hsieh CC, Wang LY (1995). Green-tea consumption and risk of stomach cancer: a population-based case-control study in Shanghai, China. *Cancer Causes and Control*.

[25] Imai K, Suga K, Nakachi K (1997). Cancer-preventive effects of drinking green tea among a Japanese population. *Preventive Medicine*.

[26] Myung SK, Bae WK, Oh SM (2009). Green tea consumption and risk of stomach cancer: a meta-analysis of epidemiologic studies. *International Journal of Cancer*.

[27] Seely D, Mills EJ, Wu P (2005). The effects of green tea consumption on incidence of breast cancer and recurrence of breast cancer: a systematic review and meta-analysis. *Integrative Cancer Therapies*.

[28] Katiyar SK, Afaq F, Azizuddin K, Mukhtar H (2001). Inhibition of UVB-induced oxidative stress-mediated phosphorylation of mitogen-activated protein kinase signaling pathways in cultured human epidermal keratinocytes by green tea polyphenol (-)-epigallocatechin-3-gallate. *Toxicology and Applied Pharmacology*.

[29] Katiyar SK, Mukhtar H (2001). Green tea polyphenol (-)-epigallocatechin-3-gallate treatment to mouse skin prevents UVB-induced infiltration of leukocytes, depletion of antigen-presenting cells, and oxidative stress. *Journal of Leukocyte Biology*.

[30] Kim J, Hwang JS, Cho YK (2001). Protective effects of (-)-epigallocatechin-3-gallate on UVA- and UVB-induced skin damage. *Skin Pharmacology and Applied Skin Physiology*.

[31] Record IR, Dreosti IE (1998). Protection by black tea and green tea against UVB and UVA+B induced skin cancer in hairless mice. *Mutation Research*.

[32] Mukhtar H, Ahmad N (2000). Tea polyphenols: prevention of cancer and optimizing health. *American Journal of Clinical Nutrition*.

[33] Middleton E, Kandaswami C, Theoharides TC (2000). The effects of plant flavonoids on mammalian cells: implications for inflammation, heart disease, and cancer. *Pharmacological Reviews*.

[34] Wu BT, Hung PF, Chen HC (2005). The apoptotic effect of green tea (-)-epigallocatechin gallate on 3T3-L1 preadipocytes depends on the Cdk2 pathway. *Journal of Agricultural and Food Chemistry*.

[35] Ahmad N, Mukhtar H (1999). Green tea polyphenols and cancer: biologic mechanisms and practical implications. *Nutrition Reviews*.

[36] Jankun J, Selman SH, Swiercz R, Skrzypczak-Jankun E (1997). Why drinking green tea could prevent cancer. *Nature*.

[37] Su LJ, Arab L (2002). Tea consumption and the reduced risk of colon cancer—results from a national prospective cohort study. *Public Health Nutrition*.

[38] Yang CS (2001). Inhibition of carcinogenesis and toxicity by dietary constituents. *Advances in Experimental Medicine and Biology*.

[39] Zhang M, Lee AH, Binns CW, Xie X (2004). Green tea consumption enhances survival of epithelial ovarian cancer. *International Journal of Cancer*.

[40] Adhami VM, Ahmad N, Mukhtar H (2003). Molecular targets for green tea in prostate cancer prevention. *Journal of Nutrition*.

[41] Hsu SD, Singh BB, Lewis JB (2002). Chemoprevention of oral cancer by green tea. *General Dentistry*.

[42] Ju J, Hong J, Zhou JN (2005). Inhibition of intestinal tumorigenesis in *Apc*
^min/+^ mice by (-)-epigallocatechin-3-gallate, the major catechin in green tea. *Cancer Research*.

[43] Lambert JD, Hong J, Yang GY (2005). Inhibition of carcinogenesis by polyphenols: evidence from laboratory investigations. *The American Journal of Clinical Nutrition*.

[44] Masuda M, Suzui M, Lim JT (2002). Epigallocatechin-3-gallate decreases VEGF production in head and neck and breast carcinoma cells by inhibiting EGFR-related pathways of signal transduction. *Journal of Experimental Therapeutics and Oncology*.

[45] Masuda M, Suzui M, Lim JT, Weinstein IB (2003). Epigallocatechin-3-gallate inhibits activation of HER-2/neu and downstream signaling pathways in human head and neck and breast carcinoma cells. *Clinical Cancer Research*.

[46] Yang CS, Maliakal P, Meng X (2002). Inhibition of carcinogenesis by tea. *Annual Review of Pharmacology and Toxicology*.

[47] Demeule M, Michaud-Levesque J, Annabi B (2002). Green tea catechins as novel antitumor and antiangiogenic compounds. *Current Medicinal Chemistry—Anti-Cancer Agents*.

[48] Maiti TK, Chatterjee J, Dasgupta S (2003). Effect of green tea polyphenols on angiogenesis induced by an angiogenin-like protein. *Biochemical and Biophysical Research Communications*.

[49] Masuda M, Suzui M, Weinstein IB (2001). Effects of epigallocatechin-3-gallate on growth, epidermal growth factor receptor signaling pathways, gene expression, and chemosensitivity in human head and neck squamous cell carcinoma cell lines. *Clinical Cancer Research*.

[50] Naasani I, Seimiya H, Tsuruo T (1998). Telomerase inhibition, telomere shortening, and senescence of cancer cells by tea catechins. *Biochemical and Biophysical Research Communications*.

[51] Boehm K, Borrelli F, Ernst E (2009). Green tea (Camellia sinensis) for the prevention of cancer. *Cochrane Database of Systematic Reviews*.

[52] Weisburg JH, Weissman DB, Sedaghat T, Babich H (2004). In vitro cytotoxicity of epigallocatechin gallate and tea extracts to cancerous and normal cells from the human oral cavity. *Basic and Clinical Pharmacology and Toxicology*.

[53] Babich H, Krupka ME, Nissim HA, Zuckerbraun HL (2005). Differential in vitro cytotoxicity of (-)-epicatechin gallate (ECG) to cancer and normal cells from the human oral cavity. *Toxicology in Vitro*.

[54] Khafif A, Schantz SP, Al-Rawi M (1998). Green tea regulates cell cycle progression in oral leukoplakia. *Head and Neck*.

[55] Liu X, Zhang DY, Zhang W (2011). The effect of green tea extract and EGCG on the signaling network in squamous cell carcinoma. *Nutrition and Cancer*.

[56] Hsu S, Lewis J, Singh B (2003). Green tea polyphenol targets the mitochondria in tumor cells inducing caspase 3-dependent apoptosis. *Anticancer Research*.

[57] Hsu S, Farrey K, Wataha J (2005). Role of p21WAF1 in green tea polyphenol-induced growth arrest and apoptosis of oral carcinoma cells. *Anticancer Research*.

[58] Xia W, Lau YK, Zhang HZ (1999). Combination of EGFR, HER-2/neu, and HER-3 is a stronger predictor for the outcome of oral squamous cell carcinoma than any individual family members. *Clinical Cancer Research*.

[59] Yu D, Hung MC (2000). Overexpression of ErbB2 in cancer and ErbB2-targeting strategies. *Oncogene*.

[60] Chang HC, Cho CY, Hung WC (2006). Silencing of the metastasis suppressor RECK by RAS oncogene is mediated by DNA methyltransferase 3b-induced promoter methylation. *Cancer Research*.

[61] Chang HC, Cho CY, Hung WC (2007). Downregulation of RECK by promoter methylation correlates with lymph node metastasis in non-small cell lung cancer. *Cancer Science*.

[62] Cho CY, Wang JH, Chang HC (2007). Epigenetic inactivation of the metastasis suppressor RECK enhances invasion of human colon cancer cells. *Journal of Cellular Physiology*.

[63] Chen PN, Chu SC, Kuo WH (2011). Epigallocatechin-3 gallate inhibits invasion, epithelial-mesenchymal transition, and tumor growth in oral cancer cells. *Journal of Agricultural and Food Chemistry*.

[64] Long NK, Kato K, Yamashita T (2008). Hypermethylation of the RECK gene predicts poor prognosis in oral squamous cell carcinomas. *Oral Oncology*.

[65] Kato K, Long NK, Makita H (2008). Effects of green tea polyphenol on methylation status of RECK gene and cancer cell invasion in oral squamous cell carcinoma cells. *British Journal of Cancer*.

[66] Shklar G, Schwartz J, Trickler D, Reid S (1989). Regression of experimental cancer by oral administration of combined alpha-tocopherol and beta-carotene. *Nutrition and Cancer*.

[67] Schwartz JL, Tanaka J, Khandekar V (1992). Beta-carotene and/or vitamin E as modulators of alkylating agents in SCC-25 human squamous carcinoma cells. *Cancer Chemotherapy and Pharmacology*.

[68] Khafif A, Schantz SP, Chou TC (1998). Quantitation of chemopreventive synergism between (-)-epigallocatechin-3-gallate and curcumin in normal, premalignant and malignant human oral epithelial cells. *Carcinogenesis*.

[69] Mohan KV, Gunasekaran P, Varalakshmi E (2007). In vitro evaluation of the anticancer effect of lactoferrin and tea polyphenol combination on oral carcinoma cells. *Cell Biology International*.

[70] Amin AR, Khuri FR, Chen ZG, Shin DM (2009). Synergistic growth inhibition of squamous cell carcinoma of the head and neck by erlotinib and epigallocatechin-3-gallate: the role of p53-dependent inhibition of nuclear factor-kappaB. *Cancer Prevention Research*.

[71] Yamamoto T, Staples J, Wataha J (2004). Protective effects of EGCG on salivary gland cells treated with gamma-radiation or cis-platinum(II)diammine dichloride. *Anticancer Research*.

[72] Moore SR, Johnson NW, Pierce AM, Wilson DF (2000). The epidemiology of mouth cancer: a review of global incidence. *Oral Diseases*.

[73] Chandra Mohan KV, Hara Y, Abraham SK, Nagini S (2005). Comparative evaluation of the chemopreventive efficacy of green and black tea polyphenols in the hamster buccal pouch carcinogenesis model. *Clinical Biochemistry*.

[74] Li N, Han C, Chen J (1999). Tea preparations protect against DMBA-induced oral carcinogenesis in hamsters. *Nutrition and Cancer*.

[75] Sugimura T (1992). Multistep carcinogenesis: a 1992 perspective. *Science*.

[76] Srinivasan P, Suchalatha S, Babu PV (2008). Chemopreventive and therapeutic modulation of green tea polyphenols on drug metabolizing enzymes in 4-Nitroquinoline 1-oxide induced oral cancer. *Chemico-Biological Interactions*.

[77] Hansel DE, Rahman A, Wehner S (2003). Increased expression and processing of the Alzheimer amyloid precursor protein in pancreatic cancer may influence cellular proliferation. *Cancer Research*.

[78] Meng JY, Kataoka H, Itoh H, Koono M (2001). Amyloid beta protein precursor is involved in the growth of human colon carcinoma cell in vitro and in vivo. *International Journal of Cancer*.

[79] Ko SY, Chang KW, Lin SC (2007). The repressive effect of green tea ingredients on amyloid precursor protein (APP) expression in oral carcinoma cells in vitro and in vivo. *Cancer Letters*.

[80] Schwartz JL, Baker V, Larios E, Chung FL (2005). Molecular and cellular effects of green tea on oral cells of smokers: a pilot study. *Molecular Nutrition and Food Research*.

[81] Li N, Sun Z, Han C, Chen J (1999). The chemopreventive effects of tea on human oral precancerous mucosa lesions. *Proceedings of the Society for Experimental Biology and Medicine*.

[82] Tsao AS, Liu D, Martin J (2009). Phase II randomized, placebo-controlled trial of green tea extract in patients with high-risk oral premalignant lesions. *Cancer Prevention Research*.

[83] Ide R, Fujino Y, Hoshiyama Y (2007). A prospective study of green tea consumption and oral cancer incidence in Japan. *Annals of Epidemiology*.

